# Combined Fainting and Psychogenic Non-epileptic Seizures as Significant Therapy Hurdles in Blood-Injury-Injection Phobia: A Mini-Review and Case Report

**DOI:** 10.3389/fpsyt.2022.915058

**Published:** 2022-07-12

**Authors:** Iven-Alex von Mücke-Heim, Isabelle Walter, Sandra Nischwitz, Angelika Erhardt

**Affiliations:** ^1^Max Planck Institute of Psychiatry, Outpatient Clinic, Munich, Germany; ^2^International Max Planck Research School for Translational Psychiatry (IMPRS-TP), Munich, Germany; ^3^Department of Psychiatry, Clinical Anxiety Research, University of Würzburg, Würzburg, Germany

**Keywords:** blood-injury-injection phobia, psychotherapy (exposure), individualization, psychogenic non-epileptic seizures, fainting, applied tension, distress tolerance skills

## Abstract

**Background:**

Anxiety disorders are the most frequent mental disorders. Among the different subtypes, specific phobias are the commonest. Due to the ongoing SARS-CoV-19 pandemic, blood-injury-injection phobia (BII) has gained wider attention in the context of large-scale vaccination campaigns and public health. In this BII phobia mini-review and case report, we describe the successful treatment of a severe BII phobia case with combined fainting and psychogenic non-epileptic seizures (PNES) and demonstrate the role of specialized outpatient care.

**Case Report:**

The patient was a 28-year-old woman. She suffered from intense fear and recurrent fainting with regard to needles, injections, injuries, and at the sight of blood since early childhood. Medical history revealed infrequent events suggestive of PNES following panic attacks after sustained exposure to phobic stimuli. Family history was positive for circulation problems and BII fears. Psychopathological evaluation confirmed BII phobia symptoms and diagnosis was made according to the DSM-5. The Multidimensional Blood/Injury Phobia Inventory short version (MBPI-K) revealed severe manifestation of the disease. Neurological examination was ordinary. Repeated electroencephalography detected no epileptic pattern. Cranial magnetic resonance imaging showed normal morphology. Treatment was carried out by a seasoned, multidisciplinary team. Cognitive behavior therapy and exposure were performed. Modification of standard treatment protocol was necessary due to hurdles posed by recurrent fainting and a severe panic-triggered dissociative PNES during *in vivo* exposure. Modification was implemented by limiting *in vivo* exposure intensity to moderate anxiety levels. In addition to applied muscle tension and ventilation techniques, increased psychoeducation, cognitive restructuring, and distress tolerance skills (e.g., ice pack, verbal self-instructions) were used to strengthen the patient's situational control during *in vivo* exposure. A total of 15 sessions were performed. Therapy success was proven by 83% reduction in MBPI-K rating, SARS-CoV-19 vaccination, and a blood draw without psychological assistance, fainting, or seizure.

**Conclusion:**

Taken together, this case demonstrates the potential of and need for specialized outpatient care and individualized treatment for severe BII phobia patients in order to provide them the perspective to have necessary medical procedures done and get vaccinated.

## Background

Anxiety disorders are the most common and second most impactful mental disorders with regards to the years lived with disability (1, 2). They account for 14% of 1-year prevalence of all psychiatric conditions (3). Lifetime prevalence estimates go as high as 33.7% (4–6). This is aggravated by the ongoing SARS-CoV-19 pandemic, which caused global anxiety disorder prevalence to increase significantly, amounting to a total of 76 million additional cases (7).

Anxiety disorders usually start during late childhood and early adolescence (median age of onset: 11 years) (4). Women have at least a 2-fold increased risk compared to men. Among the different anxiety disorder subtypes, specific phobias account for 37–67% of cases and are thus the most frequent subtype (3, 5, 8, 9). A recent meta-analysis estimated a median lifetime prevalence for specific phobias of 7.2% with a median onset of 7 years (4, 9). Pathological fear of animals (zoophobia) and of heights (acrophobia) are the most common subtypes. Specific phobias often run a chronic course (9, 10), and significantly impair quality of life and functioning of patients (11, 12). About 60.5% of specific phobia patients suffer from at least one other mental disorder during their lifetime (9, 12). Aptly, specific phobias are predictors for other psychiatric disorders including anxiety, mood and substance-use disorders (9). In a fraction of cases, anxiety disorder can also present with so-called psychogenic non-epileptic seizures (PNES) (13, 14). These seizures are paroxysmal events, that are often preceded by involuntary experience avoidance behavior (15). The latter is the avoidance of thoughts, physical sensations, and feelings that could evoke fear (15). Therefore, PNES are supposed to be not only a neuropsychiatric symptom, but an unconscious psychological defense mechanism to protect the affected individuals from unwanted and overwhelming emotions in order to maintain their intrapsychic balance (14, 16). In general, PNES are characterized by transient disturbances in sensorimotor, vegetative, and neurocognitive functions. Diagnosis should be made carefully and made based on specific criteria (i.e., typical PNES history, witnessed events, and EEG monitoring) to ensure a clear distinction of PNES from epileptic seizures. For detailed review on PNES and the diagnostic process, we would like to refer the interested reader to the works of Lafrance et al. and Baslet et al. (17–19).

The Diagnostic and Statistical Manual of Mental Disorders (DSM-5) defines specific phobias by seven criteria (Table 1) and provides clinical subtypes (20). The definition states that specific phobias are characterized by an intense, immediate, and socio-culturally out of proportion fear of a specific object or situation, namely the phobic stimulus. This fear-evoking object or situation is actively avoided or, if avoidance behaviors fail, endured with sustained fear or anxiety. To enable diagnosis, symptoms must last 6 months or longer and be severe enough to cause significant distress or sociofunctional impairment (9, 20). The novel International Classification of Diseases 11th revision, on the other hand, does not provide specific phobia subtypes due to a lack of evidence and thus validity of most phobia subtypes. However, in the case of phobia relating to blood, injury, and injection (BII), studies identified a distinct pathophysiological mechanism (9, 21–28), which we discuss in the next section.

**Table 1:** Diagnostic criteria of specific phobias.

**DSM-5 Criteria of Specific Phobia (300.29)**
A) Marked fear or anxiety about a specific object or situation (e.g., flying, animals, or blood/injection) B) The phobic object or situation almost always provokes immediate fear or anxiety C) The phobic object or situation is actively avoided or endured with intense fear or anxiety D) The fear or anxiety is out of proportion to the actual danger posed by the specific object or situation and to the sociocultural context. E) The fear, anxiety, or avoidance is persistent, typically lasting for 6 months or more. F) The fear, anxiety, or avoidance causes clinically significant distress or impairment in social, occupational, or other important areas of functioning. G) The disturbance is not better explained by the symptoms of another mental disorder […].

## Blood-Injury-Injection Phobia: A Big Obstacle in Pandemic Times

BII phobia is a unique entity featuring intense fear and frequently fainting at the sight or anticipation of blood, physical injury that could lead to blood loss, or injection (9, 28, 29). Contemporary prevalence estimates range between 2 and 4% (29–33). Median age of BII phobia onset is 6 years (10, 32). Nearly 100% of patients attain BII phobia before the age of 18 years (10). Nonetheless, studies also demonstrate BII phobia in older patients as well as in the elderly (31, 34). Aside younger age and female sex, proxies of socioeconomic status including lower income and education are common features of BII phobia patients (12, 32). From an evolutionary psychiatry perspective, the higher frequency of BII phobia in younger women is thought to be the consequence of a selection process in line with the Paleolithic-human-warfare hypothesis (35). This empirically braced theory assumes that highly danger-averse women with regard to situations and objects that potentially cause fatal blood loss have experienced a survival advantage in ancient times (9, 35). Nowadays, this advantage has faded out, especially when considering long-term health ramifications of BII phobia.

Beyond anxiety and fainting, BII phobia is an impactful and far-reaching condition. It was found to be associated with psychiatric disorders including other specific phobias and personality, mood, and anxiety disorders (10, 31, 36). It was also linked to cardiovascular complications in diabetics and elevated blood pressure in elder BII phobia patients (31, 32). For BII phobia patients, medical procedures become linked to the phobic stimulus over time (28). This phenomenon is highly problematic since modern medicine relies on needles for vaccination, blood draws, and substance application (37, 38). BII phobia furthermore runs the risk of generalization, that is a phobia of health care situations and personnel *per se* (39). Over time, these mechanisms cause a reduction or avoidance of health care services including preventive measures (9, 29, 31). In line with these findings, a contemporary review and meta-analysis by McLenon and Rogers from 2019 found that needle fear is common in patients requiring preventive health care: avoidance of influenza vaccination caused by needle fear was found in 16% of adult patients, 27% of hospital employees, 18% of employees in care facilities, and 8% of healthcare professionals in a clinical setting (40). With regard to the ongoing SARS-CoV-19 pandemic, a 2021 study from the United Kingdom in 15.014 adults found that about 10% of CoV-19 vaccine hesitancy can be explained by blood-injection-injury fears (41).

The occurrence of fainting following exposure to the phobic stimulus sets BII phobia apart, since this reaction not regularly seen in the other specific phobias (28, 29). A study by Wani et al. found fainting in 25.6% of male and in 74.4% of female BII phobia patients (29). Older studies have shown fainting rates of up to 80% (42, 43), and even reported seizures, asystolia, and death (26, 32, 39, 44). Besides intense fear and fainting, disgust plays a major role in the BII phobia reaction (45–47). In line with the unique symptomatology of BII phobia, studies have shown that the causative pathophysiological mechanism, namely a dysfunctional vasovagal reflex, runs in families (39). A recent meta-analysis demonstrated a 41% heritability for blood-related fear and 33% for BII phobia, making it the most heritable specific phobia (9, 48). Still, learning and parenting are also critically involved in the development of BII phobia (39), making it both an inherited and learned disease (49, 50). Additionally, parenting and temperament are deemed factors of BII phobia development in children and adolescents (47). Several functional neuroimaging studies have revealed dysfunction of the dorsolateral and ventromedial prefrontal cortex, the thalamus and insula, as well as the anterior cingulate and occipital cortex in BII phobia patients. These findings reflect brain regions deemed responsible for the behavioral, emotional, and neurovegetative symptoms associated with BII phobia. For instance, the cingulate and occipital cortex, along with the thalamus, and insula are known to be involved in emotional perception, which is typically disturbed in specific phobias. The insula and thalamus furthermore regulate interoception and neurovegetative functions, both commonly dysregulated in BII phobia patients. And lastly, the prefrontal and occipital cortex are involved in emotion modulation (51, 52), something that could reflect the problem of BII phobia patients to modulate their intense situational anxiety and fear. Taken together it can be reasoned, that the neuroimaging aberrations stated above map, more or less, onto BII phobia symptoms and thus embody the cornerstones of the pathophysiological brain circuits involved in BII phobia genesis.

The BII phobia pathophysiological mechanism is complex and was long considered exclusively diphasic (27), with an initial fight-or-flight response eliciting a sympathetic nervous system activation accompanied by blood pressure (BP) and hear rate (HR) increased, followed by a second phase. The latter is characterized by parasympathetic activation, resulting in a sudden BP and HR drop, cerebral hypoperfusion, and ultimately a loss of consciousness and muscle tone, also termed fainting. However, novel research indicates that only about 20% of BII patients display this diphasic response. Interestingly, these patients appear to be more severely affected than non-fainting BII patients (26, 43, 53). These findings illustrate, that BII phobia is more complex and heterogeneous than initially believed, especially concerning pathophysiological differences between fainters and non-fainters as well as among different fainting types. And, as set out above, the same is true for the diphasic hypothesis (26). Evidence for BII phobia triggered seizures is virtually non-existent (32, 54, 55).

The state-of-the-art treatment of specific phobias is exposure therapy (23, 56–58). Concerning the psychotherapy modality, the most beneficial outcomes have been reported for cognitive behavioral psychotherapy (CBT) (58), with context and stimulus variation being pivotal contributors of therapy success (56, 57). Studies indicate that *in vivo* exposure is the core therapeutic mechanism (57, 59–61). In general, exposure therapy is based on the assumption that phobic anxiety and fear are reduced by repeated exposure to feared or avoided stimuli, resulting in habituation and fear extinction by repeated violation of threat expectancy (62, 63). In this respect, two main theoretical approaches exist: according to the “emotional processing theory,” within- and between-session habituation during CBT framed exposure are deemed key in predicting fear reduction and long-term correction of cognitive mechanisms. Meanwhile, more recent approaches focus on inhibitory learning, a concept revolving around the idea that the fear stimulus memory get co-opted with a second, less threatening and context specificity memory which enables inhibition of the older stimulus memory. Inhibitory learning is currently deemed the more suitable explanation model for anxiety disorders, especially since these patients display impaired inhibitory regulation and learning and thus extinction (63). Exposure is usually performed step-by-step according to the perceived individual difficulty hierarchy, which relates to a particular exposure situation (0 = no threat expectancy, easy to handle; 100 = maximum threat expectancy, extremely difficult to handle). Therapy aims for exposure to maximum threat expectancy and fear stimuli without avoidance behavior. Several studies with different therapy and exposure modality and duration have reported favorable outcomes for BII phobia (64–67). Recently, virtual reality exposure has also proven promising (68–70). Efficacy was also demonstrated for augmented reality exposure, a similar tech-based approach, for many other specific phobias (71). Moreover, case reports have shown individuum-level success for approaches like self-exposure (67), individualization of therapy (72), cognitive restructuring (73), and applied tension (74). In addition to *in vivo* exposure, applied muscle tension, and breathing techniques are commonly employed to reduce vagal activation, anxiety-related respiratory dysregulation, and thus fainting (23, 27, 45, 66, 75–77). A recent study used a simulated blood draw paradigm and demonstrated that applied muscle tension resulted in significant elevation of cerebral oxygenation and end-tidal CO^2^ partial pressure. Overall, but especially in a time critical setting, applied muscle tension seems to be more effective than breathing techniques (78). Also, longer therapy session regimens seem to generate more favorable outcomes compared to shorter ones, with sustained exposure appearing to be the key factor of long-term treatment success (57, 61). However, current evidence concerning the optimal number of therapy sessions is heterogeneous and does not allow for peremptory conclusions (23, 79). Still, the core of these findings aligns with the fact, that exposure therapy is the single most effective treatment for all specific phobias and that multiple therapy sessions are highly effective in terms of sustained improvement (61).

The presented evidence highlights the complexity and importance if BII phobia, especially in respect to the ongoing SARS-CoV-19 pandemic. In the present article, we report the successful treatment of a severe case of BII phobia in a young woman with fainting and panic-triggered probable PNES by use of modified exposure therapy, distress tolerance skills, applied tension, and breathing techniques. As of today, this is the first published case report on the treatment of combined fainting and panic-triggered non-epileptic seizures in BII phobia.

## Case Presentation

The patient first presented herself in our specialized outpatient clinic at the age of 28 with the wish to get treated for severe anxiety and panic as well as fainting related to blood, injuries, and injections. Symptoms were present since childhood and worsened over time. The fear of fainting and the fear of pain during insertion of the needle were the patient's main focus. The patient was highly motivated, since she wanted to reduce anxiety and get vaccinated for SARS-CoV-19 to avoid potential health complications and obstacles in daily life.

The patient was of European descent, unmarried but in a stable relationship without children. Since graduating university, she was employed as a Sales Manager. She suffered from BII related fear and anxiety and fainting (usually <30-second-long loss of conscious, no motor symptoms, instant reorientation, max. once per month) and from very infrequent non-epileptic seizures due to anxiety and panic attacks in situations of sustained exposure to blood-related phobic stimuli or unexpected pain (e.g., needles, pain, swallowing hot food). Typical epileptic stimuli like alcohol consumption or sleep deprivation had not yet triggered a single seizure event. One time, observers allegedly noticed myoclonic jerks during a fainting in school. A neurological diagnostic confirmation in the course of a hospital stay during childhood yielded no epileptic origin of the recurrent fainting and seizures. Medical history also showed hypothyroidism diagnosed at the age of 23, migraine, and allergies including pollinosis and atopic eczema. Hypothyroidism was treated by daily administration of 75 μg L-Thyroxin. At the age of 12, the patient once consulted a psychologist for BII-related fears, but no long-term treatment was initiated. Family history revealed circulation problems and needle fear in siblings. The patient's birth and development were ordinary. Vaccination and routine blood draw were last performed during late childhood.

Disease-specific anamnesis revealed that the patient was unable to remember a time without intense fear of needles. At the age of 2, she forcibly removed an intravenous catheter in the course of a tonsillectomy while struggling against the procedure and suffering a panic attack. The first fainting happened in primary school after falling down a flight of stairs. At the age of 11, she had first fainted in the context of a vaccination. As a result, the fear of injections was reinforced, and combined with the fear of fainting following injections. During the anticipation of blood donation in adolescence, she fainted again and fell. She suffered a cerebral contusion. BII fears negatively influenced hobbies (e.g., skiing) and her stand on pregnancy and childbirth.

Self-reporting psychometry was performed prior to the treatment by German versions of the Patient-Health-Questionnaire (PHQ-D) (80), the Beck-Depression-Inventory II (BDI-II) (81), the Panic- and Agoraphobia Scale (PAS) (82), the Yale-Brown Obsessive-Compulsive Scale (83), the Liebowitz Social Anxiety Scale (LSAS) (84), the ADHD self-rating scale (ADHS-SB) (85), the Childhood Trauma Questionnaire (CTQ) (86), and the Relationship Scales Questionnaire (RSQ) (87), and the Assessment of DSM-IV Personality Disorders (ADP-4). Unfortunately, the ADP-4 results could not be analyzed and reported, since the patient did not answer several questions. Moreover, the Multidimensional Blood/Injury Phobia Inventory short version (MBPI-K) was assessed before and after the treatment. The MBPI-K consists of 20 items that are rated on a 5-point scale and includes four fear dimensions (blood, fainting, needles, and hospital). The questionnaires used are standardized and established in the field. In case of the comparatively novel MBPI, validation of the German version has been performed in 2010 demonstrating high consistency and construct validity along with good reliability and validity for both the long (MBPI) and short (MBPI-K) version. The MBPI is the gold standard of BII phobia assessment (88). The PHQ-D revealed low generalized anxiety (Generalized Anxiety Disorder 7 Scale) but panic symptoms (PHQ-Panic) limited to BII situations. The pre-treatment MBPI-K revealed the following values in the four scales: 20 out of 20 points for needle fears, 11.25 out of 20 points fainting, 15 out of 20 points for blood and injury fears and disgust, 15 out of 20 for hospital fears (sum value: 61.25). The LSAS showed low levels of social fear and avoidance and the PAS indicated sub-diagnostic agoraphobic fears. The RSQ revealed a secure attachment style (87, 89). In direct comparison to a non-clinical control sample (87), RSQ subscale values showed very low anxiety levels with regard to relationship breakup and intimacy, low values for lack of trust, and an overall high independency desire. All other questionnaires showed no clinically relevant results.

At the first presentation in our clinic, the diagnosis of BII phobia according to the DSM-5 was confirmed based on psychopathological assessment. No other comorbid psychiatric disorders were detected. Anticipation of BII-associated stimuli caused anxiety symptoms including palpitations, sweating, shaking, dizziness, and avoidance. Moreover, the patient suffered from recurrent fainting, an intense fear of fainting, and infrequent seizure events. Triggers for fainting included sight of blood, anticipation of injury or injection, and non-invasive medical procedures like palpation. Seizures were very infrequently evoked by injections and injuries. Based on semiology and patient history, we suspected the reported infrequent seizures events to be, most likely, of non-epileptic, psychogenic origin.

The entire treatment process was performed in a specialized outpatient setting, which is tailored to the specific needs of anxiety disorder patients in general and BII phobia ones in particular. Therapies were performed primarily by a psychological psychotherapist and a senior and junior psychiatrist, which were supported by a seasoned and multidisciplinary team composed of a neurologist, a (neuro)radiologist, and a primary care physician. CBT was performed in four phases according to an established, standardized German therapy manual for BII phobia by Schienle and Leutgeb (90). The treatment consisted of 15 sessions and each session lasted about 25–90 min (Table 2). The first phase was primarily characterized by psychoeducation. Here, the patient learned about the BII phobia as an anxiety disorder including its aetiopathogenesis and perpetuating mechanisms. Further topics were emotional stress like anxiety, fear, and disgust in the context of fainting. Feelings of shame were not present. The patient was in constant exchange about her symptoms and therapy progression with friends and family, indicating a strong social network. She reported psychoeducation to be very helpful to accept her disturbing symptoms. The second treatment phase consisted of the preparation of the graduated exposure procedure including the training of the applied tension and breathing techniques to counterbalance the second part of the diphasic response to reverse the drop in HR and BP and thus loss of consciousness and fainting. Also, phobic stimuli were translated into the following individual difficulty hierarchy: finger prick (29), watching videos of the procedure of a blood collection and the application of an intravenous catheter (49), a real syringe (49), getting vaccinated (64), and having blood drawn (89). During the third phase of treatment *in sensu* exposure, meaning exposure only by use of imagination and image-, sound-, or video-based techniques, was administered with gradual increase according to the individual difficult hierarchy (e.g., watching needle-related and blood draw videos). During the first *in sensu* exposure, the patient rated her maximum level of fear by 8/10 on a numeric rating scale (NRS) and reported palpitations. In the fourth and last phase of the treatment, *in vivo* exposure was administered gradually by finger prick with a lancet and, after successful completion, repeated venous blood draws from superficial forearm veins. During finger pricks, fainting occurred during the first out of three separate finger prick exposures. At the first blood draw exposure, the patient rated her maximum anxiety level 10/10 in the NRS accompanied by an increased heart rate, internal tension and weeping. Here, the patient successfully used applied tension and was advised to employ anti-vagal breathing techniques, i.e., normal frequency breathing, and to avoid Valsalva maneuverers like breath holding or abdominal prelum. The third and fourth phase also entailed cognitive restructuring to change maladaptive beliefs and threat expectancies.

**Table 2:** Individual therapy sessions including duration and main therapeutic interventions.

**Therapy session no./time point**		**Duration (min)**	**Main diagnostics and therapeutic interventions**
1	Therapy week 1	90	First consultation and medical history (anamnesis) Psychometry (questionnaires incl. MBPI-K) Clinical examination
2	Therapy week 2	60	Psychoeducation: information about the treatment and diagnosis of BII phobia
3	Therapy week 3	60	Psychoeducation: therapy goal, aetiopathogenesis and perpetuating mechanisms of BII phobia, pathophysiology of fainting, applied muscle tension training
4	Therapy week 4	60	Psychoeducation: information about the pending exposure, individual difficulty hierarchy of phobic stimuli
5	Therapy week 5	90	*In sensu* exposure: watching videos of a blood-draws, cognitive therapy (restructuring) Self-administered *in sensu* exposure (repeatedly): watching videos at home between sessions five and six
6	Therapy week 6	70	Repeated *in vivo* exposure: finger prick (3x), short fainting (few seconds) Self-administered *in vivo* exposition (repeatedly): finger prick between sessions six and seven
7	Therapy week 7	90	*In vivo* exposure: blood draw
8	Therapy week 8	150	*In vivo* exposure: blood draw, panic attack, seizure (PNES) Electroencephalography, thorough clinical examination
9	Therapy week 9	90	Debriefing on the last *in vivo* exposure, psychoeducation about the PNES, training of distress tolerance skills and verbal self-instructions, cognitive therapy (restructuring)
10	Therapy week 10	90	Modified *in vivo* exposure: blood draw Cognitive therapy (restructuring)
11	Therapy week 10	60	Modified *in vivo* exposure: blood draw Cognitive therapy (restructuring)
12	Therapy week 11	60	Modified *in vivo* exposure: blood draw Cognitive therapy (restructuring)
13	Therapy week 11	60	Modified *in vivo* exposure: blood draw (patient watches the needle insertion) Magnetic resonance imaging 1.5 Tesla (neurocranium) Electroencephalography
14	Therapy week 12	60	Self-initiated blood draw under real world conditions (no psychological assistance, final *in vivo* exposure) Transfer of coping strategies to daily life MBPI-K questionnaire
15	Therapy week 13	60	Summary and completion of therapy (catamnesis)

During the second blood draw exposure in the eighth therapy session, the patient reported increased anxiety and presyncope symptoms indicative of vasovagal overactivation. Initially, the patient was able to use applied tension and stop her tachypnoea and Valsalva maneuvers under direct therapeutic advisement. However, after the insertion of the needle, she reported anxiety and presyncope symptoms and again involuntarily restarted Valsalva maneuvers. These symptoms could not be countered by applied tension, breathing techniques, and passive leg-raising. The patient then suddenly reported extreme anxiety and suffered a panic attack. The needle was instantly removed. The panic attack then changed into a seizure. The latter was accompanied by a loss of consciousness and its duration was short (~2 min). Eyes were closed to about 80% with no detectable bulbus deviation. Limb and trunk posture were highly tonic and suggestive of opisthotonos. Lips and perioral areas were briefly and mildly cyanotic. Both hands showed an incomplete Trousseau sign of latent tetany and her arms made slow and asynchronous waxing movements. No convulsions were visible. Involuntary urination but no defecation or tongue bite occurred. Directly after the motor symptoms had terminated, the patient uttered repetitive phrases (e.g., “oh no”). Reorientation and recurrence of fear and anxiety occurred immediately after the seizure. Confusion was limited to <3 min after the event. The instantly performed clinical neurological examination was ordinary. Memory loss was restricted to the seizure itself. Same-day electroencephalography showed normal Alpha-activity with intermittent unspecific arrhythmic activity. No interictal epileptic discharges, abnormal slowing, or any other focal abnormalities could be detected. Cranial 1.5 Tesla magnetic resonance imaging (MRI) showed no pathological result. Another electroencephalography with activation by hyperventilation 3 weeks later also revealed normal results without any sign of epilepsy (in particular absence and temporal lobe epilepsy) (91–93). The overall clinical presentation and diagnostic results were highly suggestive of a PNES, namely a panic-triggered conversion symptom.

Following the diagnosis of probable PNES (present criteria: typical PNES history, characteristic seizure witnessed by a clinician, no epileptiform activity in routine EEG), which was based on the diagnostic requirements of the *International League Against Epilepsy, Non-epileptic Seizures Task Force* from 2013 (18), the standard rationale of the CBT-based exposure was modified and tailored to the patient's individual psychological situation. Forth on, instead of provoking maximum anxiety, a reduction of the emotional and neurovegetative arousal caused by the phobic stimulus was the primary treatment objective. First, we identified the level of emotional arousal that was likely leading to a panic-triggered PNES. Next, the patient was trained in distress regulation skills using a porcupine ball, an ice pack, and verbal self-instructions. Exposure was dialed back to finger pricks in order to train the newly acquired distress tolerance skills. Furthermore, the patient again trained her awareness for early signs of fainting to use the applied tension technique. We then successfully continued the *in vivo* exposure. Duration of a blood draw was prolonged up to 14 min to allow habituation and extinction learning. At the sixth and last in-therapy blood draw exposure, the patient rated her maximum anxiety level with 3.5/10 in the NRS. No more fainting or PNES occurred after modification of CBT-based exposure rationale.

After the last treatment session, the patient reported a marked reduction of fear of needles, injections, injuries, seeing blood and of fainting as well as of reduced avoidance behaviors. The post-treatment MBPI-K revealed the following values in the four scales: four out of 20 points for needle fears, one out of 20 points fainting, 3.5 out of 20 points for blood and injury fears and disgust, two out of 20 for hospital fears (sum value: 10.5). Overall, the MBPI-K reduced by 83% due to the treatment process. Further signs of therapy success were visiting her grandmother in the hospital, the ability to look at pictures of needles, and her two vaccinations against SARS-CoV-19, and a self-initiated blood draw (anxiety in the NRS maximum 5/10) in the course of a scientific study without psychological assistance, fainting, or seizure. Last but not least, the patient reported a significant increase in quality of live.

## Discussion

The present case report highlights, that severe BII phobia symptoms like recurrent fainting and panic-triggered PNES pose significant therapy obstacles, but can be overcome by modified exposure therapy in combination with applied tension and breathing techniques. Remarkably, despite modification of treatment protocol in the form of avoidance of sustained maximum anxiety during exposure, therapy was successful. This individualized approach, additional to the specialized, multidisciplinary outpatient setting and broad psychometry, can be considered the main strengths of this case report. Overall therapy success was made possible by combined use different approaches, foremost counterbalancing negative effects of in-therapy avoidance by increased psychoeducation and additional cognitive restructuring, elevated situational control during *in vivo* exposure by distress tolerance skills and applied tension, patient characteristics like low levels of social fear and avoidance in the LSAS, high therapy motivation as indicated by the desire for independency in the RSQ, an ostensible strong therapist-patient relationship made possible by the secure attachment style found in the RSQ (89), no psychiatric comorbidities in the PHQ-D or any of the other diagnostic questionnaires, and context variation during exposure (i.e., different *in sensu* and *in vivo* self-exposure by herself, the psychotherapist, and different physicians). These factors overlap with a recent analysis of predictive markers for positive therapy outcome (56). Disgust, which is also deemed a positive factor, played a minor but still contributory role in psychoeducation, cognitive restructuring, and exposure (45, 46, 56).

Our patient presented with typical features of vasovagal and orthostatic fainting, respectively, during *in vivo* exposure (finger prick), which is very common in female BII phobia patients (29, 42, 43). Our patient also suffered a single seizure during a blood draw exposure. The latter must be considered rather exceptional, since published evidence on seizures in BII is very rare. In our case, the potential explanation for the seizure can be a physical (i.e., atypical and/or convulsive syncope, epilepsy), psychiatric (i.e., conversion or dissociation phenomena masked as a panic-triggered PNES), or a combined one. A recent review by Brown and Reuber (94) argues, that PNES are heterogeneous and subsume many aetiological mechanisms including an anxiety- and panic-related genesis of conversion or dissociation phenomena. Since the neurological work-up of the present case *via* cranial MRI and repeated EEG monitoring revealed no pathological results and seizure occurrence was limited to BII phobia related stimuli, we argue that the seizure was likely a panic-triggered dissociative event in the form of a PNES. As stated earlier in the manuscript, the diagnostic certainty of the PNES in this case must be considered probable (18), more so because many of the patient's seizure features overlap with the PNES diagnostic criteria. Meanwhile, findings are insufficient and unsuitable for the diagnosis of an epileptic event or convulsive syncope (55, 95, 96). The hypothesis of a panic-triggered PNES is further corroborated by the patient's medical history, the seizure semiology, and the exclusively BII-related seizure triggers. In accordance with the dissociative and conversion aspects of PNES genesis (94, 97), we argue that the patient most likely suffered a conversion symptom in the form of a PNES with both physical as well as dissociative psychiatric symptoms (loss of consciousness, memory loss for seizure event) to escape the unexpected, maximum anxiety and loss of control. However, it has to be noted, that loss of consciousness and memory are not specific for PNES, since they can also occur in other paroxysmal conditions (e.g., syncope) (98). They are no nosological entity on their own, but rather the symptom of an underlying psychiatric disorder or condition, as we have already described earlier (99). In the present case, we believe that the singular PNES was a symptom related to the severity of the anxiety evoked by the BII phobia.

In general, conversion symptoms can occur if a patient is unable to cope with acute psychosocial stress or an immense internal psychological conflict, which causes signs and symptoms indicative of but inconsistent with known disorders, here epilepsy (97, 100). As with anxiety disorders, the most effective treatment for conversion symptoms is CBT focussing on the genesis of physical symptoms and loss of consciousness as well as individual emotional capacities (100). In the present case, we employed highly individualized CBT with increased psychoeducation and cognitive restructuring along with a modified exposure rationale and dialectical behavior therapy derived distress tolerance skills to enable treatment success.

Aside many strengths, our case report has some limitations. First, the specialized outpatient setting, in which treatment was performed by a multidisciplinary team over the course of many sessions, must be considered different from regular care. This means, that available time and personnel are comparatively high. Second, though patient characteristics match many of the typical BII phobia demographic criteria (10, 12, 29, 32), severity was very high and combined with probable panic-triggered PNES. Both, the specialized outpatient setting and individual patient characteristics, limit generalizability of our findings to average health care and BII phobia cases. Analogously, the psychotherapy focus of our specialized setting is CBT, which limits generalisability of findings to other psychotherapy modalities. Third, it needs to be mentioned, that the PNES diagnosis was made without capturing a typical seizure during a continuous EEG recording, which only allows to class the diagnostic certainty as probable. Lastly, though the present case demonstrates high effectivity on an individual level, additional research is needed to check if therapy success can be replicated, the *conditio since quam non* for generalizing our findings.

## Conclusion

Altogether, our case report highlights that severe BII phobia with combined fainting and panic-triggered PNES can be treated effectively by using individualized and modified CBT-framed exposure and psychological as well as physical control measures like distress tolerance skills and applied tension. In the future, institutions treating similar patients to the one described in this case report should aim to adapt recommendations from randomized clinical trials and guidelines related to BII phobia and PNES to provide optimal treatment in the form of *a priori* treatment individualization. Ultimately, considering the ongoing SARS-CoV-19 pandemic and long-term implications of a non-vaccinated status, our case also makes abundantly clear that therapy success and vaccination can be made possible even for the most severe BII phobia cases.

## Data Availability Statement

The original contributions presented in the study are included in the article/supplementary material, further inquiries can be directed to the corresponding author.

## Ethics Statement

Written informed consent was obtained from the individual(s) for the publication of any potentially identifiable images or data included in this article.

## Author Contributions

I-AM-H and IW reviewed the scientific literature and wrote the manuscript. SN contributed neurological and scientific expertise and aided restructuring of the manuscript. AE reviewed and edited the manuscript and provided scientific advisement. All authors contributed to the article and approved the submitted version.

## Funding

I-AM-H received funding from the International Max Planck Research School for Translational Psychiatry.

## Conflict of Interest

The authors declare that the research was conducted in the absence of any commercial or financial relationships that could be construed as a potential conflict of interest.

## Publisher's Note

All claims expressed in this article are solely those of the authors and do not necessarily represent those of their affiliated organizations, or those of the publisher, the editors and the reviewers. Any product that may be evaluated in this article, or claim that may be made by its manufacturer, is not guaranteed or endorsed by the publisher.

## Abbreviations

ADHS-SB: ADHD self-rating scale
ADP-4: Assessment of DSM-IV Personality Disorders
BDI-II: Beck-Depression-Inventory II
BII: Blood-injury-injection
BP: Blood pressure
CTQ: Childhood Trauma Questionnaire
CBT: Cognitive behavioral therapy
DSM-5: Diagnostic and Statistical Manual of Mental Disorders
HR: Heart rate
LSAS: Liebowitz Social Anxiety Scale
MBPI/-K: Multidimensional Blood/Injury Phobia Inventory / short version
MRI: Magnetic resonance imaging
NRS: Numeric rating scale
PAS: Panic- and Agoraphobia Scale
PHQ-D: Patient-Health-Questionnaire
PNES: Psychogenic non-epileptic seizure(s)
RSQ: Relationship Scales Questionnaire
SARS-CoV-19: Severe acute respiratory syndrome coronavirus type 2.
